# Resetting of Auditory and Visual Segregation Occurs After Transient Stimuli of the Same Modality

**DOI:** 10.3389/fpsyg.2021.720131

**Published:** 2021-09-21

**Authors:** Nathan C. Higgins, Ambar G. Monjaras, Breanne D. Yerkes, David F. Little, Jessica E. Nave-Blodgett, Mounya Elhilali, Joel S. Snyder

**Affiliations:** ^1^Department of Psychology, University of Nevada Las Vegas, Las Vegas, NV, United States; ^2^Department of Electrical and Computer Engineering, Johns Hopkins University, Baltimore, MD, United States

**Keywords:** auditory-visual perception, stream segregation, psychophysics, sensory distractors, auditory scene analysis, visual scene analysis

## Abstract

In the presence of a continually changing sensory environment, maintaining stable but flexible awareness is paramount, and requires continual organization of information. Determining which stimulus features belong together, and which are separate is therefore one of the primary tasks of the sensory systems. Unknown is whether there is a global or sensory-specific mechanism that regulates the final perceptual outcome of this streaming process. To test the extent of modality independence in perceptual control, an auditory streaming experiment, and a visual moving-plaid experiment were performed. Both were designed to evoke alternating perception of an integrated or segregated percept. In both experiments, transient auditory and visual distractor stimuli were presented in separate blocks, such that the distractors did not overlap in frequency or space with the streaming or plaid stimuli, respectively, thus preventing peripheral interference. When a distractor was presented in the opposite modality as the bistable stimulus (visual distractors during auditory streaming or auditory distractors during visual streaming), the probability of percept switching was not significantly different than when no distractor was presented. Conversely, significant differences in switch probability were observed following within-modality distractors, but only when the pre-distractor percept was segregated. Due to the modality-specificity of the distractor-induced resetting, the results suggest that conscious perception is at least partially controlled by modality-specific processing. The fact that the distractors did not have peripheral overlap with the bistable stimuli indicates that the perceptual reset is due to interference at a locus in which stimuli of different frequencies and spatial locations are integrated.

## Introduction

Our sensory systems are continuously tasked with extracting the most relevant information from noisy environments. The process of perceptual decision making requires integrating and segregating stimulus features into coherent streams to determine which to bring to the forefront of perception. These perceptual decisions when given ambiguous information require time to build up, after which a percept can be maintained, or superseded by an alternate percept, akin to a balancing act between perceptual stability and sensitivity to newer, more relevant information (Snyder et al., 2015). The underlying neural substrate for this bistable process is a combination of adaptation, inhibition, and noise in the sensory pathway, all exerting influence on neural representations of alternating percepts (Rankin et al., 2015; Little et al., 2020).

Bistable, ambiguous stimuli that elicit spontaneous switches between integrated and segregated percepts provide a useful tool for studying how sensory systems prioritize information. Established stimulus parameters that provoke equivalent integrated/segregated perceptions, though bistable, also have a strong initial bias to be perceived as integrated before switching to segregated, and introduction of transient stimuli of the same sensory modality typically “reset” perception from segregated to integrated (Anstis and Saida, 1985; Rogers and Bregman, 1998; Cusack et al., 2004; Roberts et al., 2008). These dynamics suggest that the neural mechanism responsible for making perceptual-decisions has a default, or baseline percept, and prompts the question of whether the same switching mechanisms are responsible for spontaneous switches as stimulus driven switches, and whether this process operates as a modality-general network as predicted by Global Workspace Theory (Changeux and Dehaene, 2008; Dehaene and Changeux, 2011).

To answer some of these questions, we tested the effectiveness of same- vs. different-sensory modality distractors to disrupt perceptual segregation. Two experiments were conducted, one with auditory stimuli as the primary task (ABA-experiment) and one with visual stimuli as the primary task (Plaid-experiment). Each experiment used a bistable stimulus capable of eliciting mutually exclusive percepts corresponding to an integrated, or segregated pattern, and the effect of occasional distractors of the same, or different sensory modality was assessed.

The ABA streaming and moving plaid stimuli represent two analogous paradigms in the auditory and visual domains, respectively (Pressnitzer and Hupé, 2006; Kondo et al., 2012a, 2018; Denham et al., 2018). ABA streaming experiments often consist of repeated tone triplets in a low-high-low-blank (ABA_) configuration, alternately perceived as a single integrated auditory stream described as “galloping,” or two segregated auditory streams described as “two metronomes” (Van Noorden, 1975; Bregman, 1990). The moving plaid paradigm consists of superimposed gratings that move at a consistent speed and are alternately perceived as an integrated moving object, or as two segregated objects “moving outward” (Wuerger et al., 1996; Hupé and Rubin, 2003).

Behaviorally these two paradigms share a number of important features, notably that perception switches between a single coherent stream to segregated streams of information. Both paradigms also tend to follow a similar initial response pattern where the first perceptual phase is integrated for a relatively long time, followed by switching back-and-forth between shorter duration segregated and integrated percepts, supporting the hypothesis that the integrated percept serves as the default, or baseline, given stimulus parameters that elicit bistable perception (Hupé and Rubin, 2003; Pressnitzer and Hupé, 2006; Li et al., 2019). Additional hallmarks of spontaneous bistable perception, such as unpredictability from one perceptual phase to the next, and observance of logarithmic distribution of phase durations, are also present in both the ABA and moving plaid streaming paradigms (Pressnitzer and Hupé, 2006; Carter et al., 2014; Denham et al., 2018).

## Methods

### Participants and Experimental Setup

Thirty-five normal hearing adults (23 female) with average age of 24.5 years (range: 18–44) participated in the ABA-experiment. Twenty-six normal hearing adults (24 female) with average age of 25 years (range: 18–45) participated in the Plaid-experiment. There was no overlap in participant pool between experiments. All participants were recruited from the community in and around the University of Nevada, Las Vegas. Prior to experimental procedures, all participants provided informed consent and answered demographic questionnaires. All techniques and procedures were approved by the University of Nevada, Las Vegas, Internal Review Board. Prior to the experiment, all participants were screened to ensure normal audiometric thresholds (<25 dB hearing levels tested at 0.25, 0.5, 1, 2, 4, and 8 kHz). An additional 6 participants in the ABA-experiment, and 12 participants in the Plaid-experiment were recruited, but not included in the data analysis due to technical problems, history of head trauma, or hearing thresholds at one or more of the tested frequencies outside the acceptable range. A priori power analyses were performed using G^*^Power, indicating that each experiment required 20 participants for the main effect of condition and 25 participants for the condition-by-starting percept interaction, for a large effect size of $\eta_{p}^{2}$ = 0.4 with 80% power.

All auditory and visual experimental stimuli were presented using Presentation (Neurobehavioral Systems) and sound stimuli generated using Matlab (Mathworks). The ABA-experiment was conducted in combination with electroencephalography (EEG; data not shown) and auditory stimuli were delivered via E-A-RTONE 3A Insert Earphones at 65 dB SPL. In the Plaid-experiment, sound was delivered via over-ear Sennheiser HD 280 headphones also at 65 dB SPL. Participants were seated in a sound attenuation chamber in front of a computer monitor in both experiments.

### ABA-Experiment: Stimulus Presentation and Protocol

**Figure 1:**
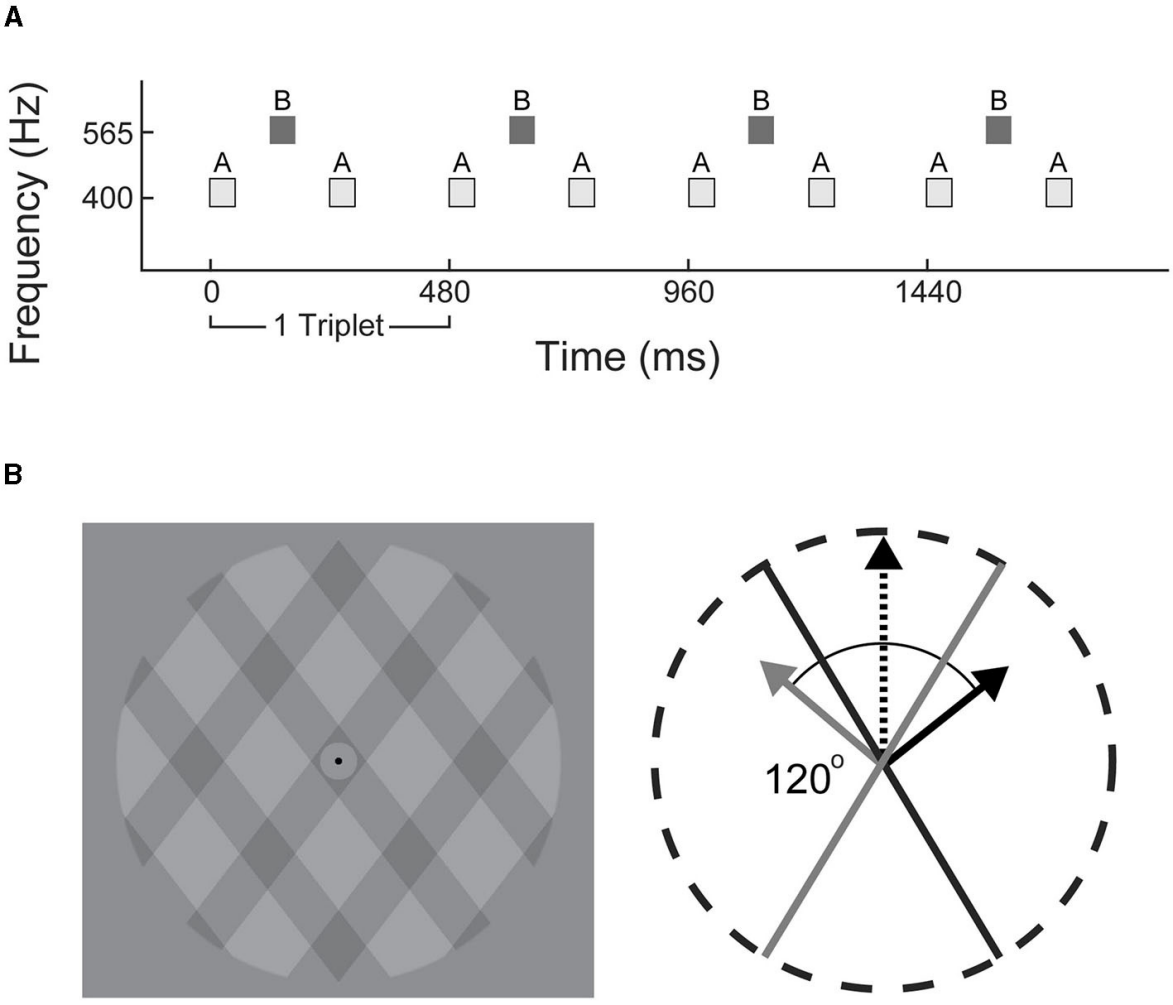
Stimulus schematics. **(A)** Experiment 1: triplets of tones in low-high-low (ABA_) configuration were used to generate alternating integrated (ABA) or segregated (A-A–, -B–) percepts. **(B)** Experiment 2: visual plaid paradigm consisted of translucent grids overlaid at 120° angle. Each frame incrementally shifted at a rate of 60 Hz to generate apparent motion in an integrated upwards (black dashed arrow), or segregated outwards direction (solid gray and black arrows).

Stimuli consisted of repeating ABA_ triplets, where A (400 Hz) and B (565.5 Hz) were pure tones and the blank_ represents a missing B tone (ABA for shorthand; illustrated in Figure 1A). The A and B tones were 50 ms each with 120 ms between the onset of each tone. Each triplet was 480 ms duration; 100 uninterrupted triplets made up each trial (total duration of 48 s). Eight consecutive trials with a 5 s rest in between defined an experimental block. Three blocks of only auditory distractors and three blocks of only visual distractors were presented in alternating order. The distractor type in the first block of the experiment was counter-balanced across participants. Auditory and visual distractors were jittered relative to the onset of a triplet with delays of 60, 180, 300, and 420 ms pseudo-randomly placed throughout the experiment; no distractors were presented over the first 20 triplets (9.6 s) of each trial. Each trial contained eight distractors, with a minimum of six triplets (2.88 s) separation between distractors. Each experiment presented 384 total distractors.

Participants were instructed to continuously indicate their perception by holding down button 1 (integrated) or button 2 (segregated) on a button box (located side by side). Prior to the experiment, participants engaged in practice trials that included the distractors. They were not specifically instructed to ignore or attend to these stimuli, but to report their perception regardless of other sounds and visual flashes on the fixation screen.

### Plaid-Experiment: Stimulus Presentation and Protocol

All visual stimuli were presented on a 24-inch monitor (60 Hz) and auditory distractors were delivered via over-ear headphones (Sennheiser HD 280). The plaid stimulus (Figure 1B) was modified from a previous study (Carter et al., 2014) and consisted of rectangular-wave gratings with a duty cycle of 0.33 (one third dark gray, two thirds light gray), with each dark/light-gray period spanning ~1 degree of visual angle. The regions where the dark gray gratings intersected were visibly darker, contributing to the perception of a single bound plaid surface, or two transparent gratings with the left- or right-angled gratings layered on top. The plaid pattern was presented within a circular aperture with a gray background. A black fixation point was located at the center. Apparent movement was generated by shifting the dark gratings incrementally in a series of 60 images over the course of 1 s (60 Hz) corresponding to a speed of 2 degrees per second.

The moving plaid stimuli were continuously presented over 48 s trials. Eight consecutive trials with a 5 s rest in between defined an experimental block. Three blocks of only auditory distractors and three blocks of only visual distractors were presented in alternating order. The distractor type in the first block of the experiment was alternated subject-to-subject. The temporal dynamics of distractor presentation within trials was very similar to the ABA-experiment. Auditory and visual distractors were jittered relative to the first image in the plaid sequence, with delays of 60, 180, 300, and 420 ms pseudo-randomly placed throughout the experiment; no distractors were presented during the first 10 s of each trial. Each trial contained eight distractors, with a minimum of 3 s between distractors. Each experiment presented 384 total distractors. Participants were instructed to maintain fixation throughout the experiment and continuously indicate whether the gratings were perceived as integrated (moving vertical) or segregated (moving horizontally) by holding down button 1 (integrated) or button 2 (segregated) on a button box.

### Distractor Stimuli

Similar auditory and visual distractors were presented in ABA- and Plaid-experiments. Auditory distractors consisted of 500 ms iterated rippled noise centered at 1, 2, or 3 kHz and were presented concurrently with the ABA stimuli with relative delays as specified above. An iterated ripple noise is defined by a noise signal *y*_0_(*t*), which is recursively summed with time-shifted versions of itself, as follows.


$$\begin{array}{l} y_{n}\left(t\right)=y_{n-1}\left(t\right)+y_{n-1}\left(t-\frac{1}{f}\right) \end{array}$$

The value of *f* determines the frequency of the resulting stimulus. We used a total of seven iterations [*y*_7_(*t*)]. This stimulus was selected for its highly salient character, a jarring sound that is difficult to ignore. This stimulus was high-pass filtered above 1 kHz, using a 5th order Butterworth filter to ensure that the spectral energy from the distractor stimuli had negligible overlap in frequency with the ABA tones. Finally, a 50 ms cosine ramp was applied to the beginning and end of the stimulus. Visual distractors consisted of a 500 ms change in color (red, green, or blue) of the fixation screen (ABA-experiment) or background surrounding the plaid-aperture (Plaid-experiment).

### Data Analysis: Phase Duration

Duration of perceptual phases was calculated as the amount of time between alternating button presses (switches). In an effort to avoid bias as a result of a relatively short continuous stimulus presentation duration (48 s per trial), phase-duration estimates that lasted the entirety of the trial were excluded from analyses focused on the initial perceptual phase duration.

### Data Analysis: Distractor-Induced Switches

The response time-course for each 48 s trial was segmented into 100 ms bins, with each bin designated as a switch or no-switch. The response time window was designated from 300 to 2,000 ms following the onset of a distractor. No-distractor time-courses were comparably defined as 300–2,000 ms following the sound onset of a triplet; note that constraints were put in place to avoid overlapping “no-distractor” time-windows and the presentation of distractors. Each of these response time-windows, or analysis epochs were designated by their initial perceptual state as integrated or segregated. Further designations were made for switch or no-switch, with an additional designation of switch direction: a switch from integrated to segregated, or segregated to integrated. Analysis epochs were also defined by distractor type: auditory (1, 2, or 3 kHz iterated rippled noise), or visual (red, green, or blue screen flash).

## Results

### Bistable Perception

**Figure 2:**
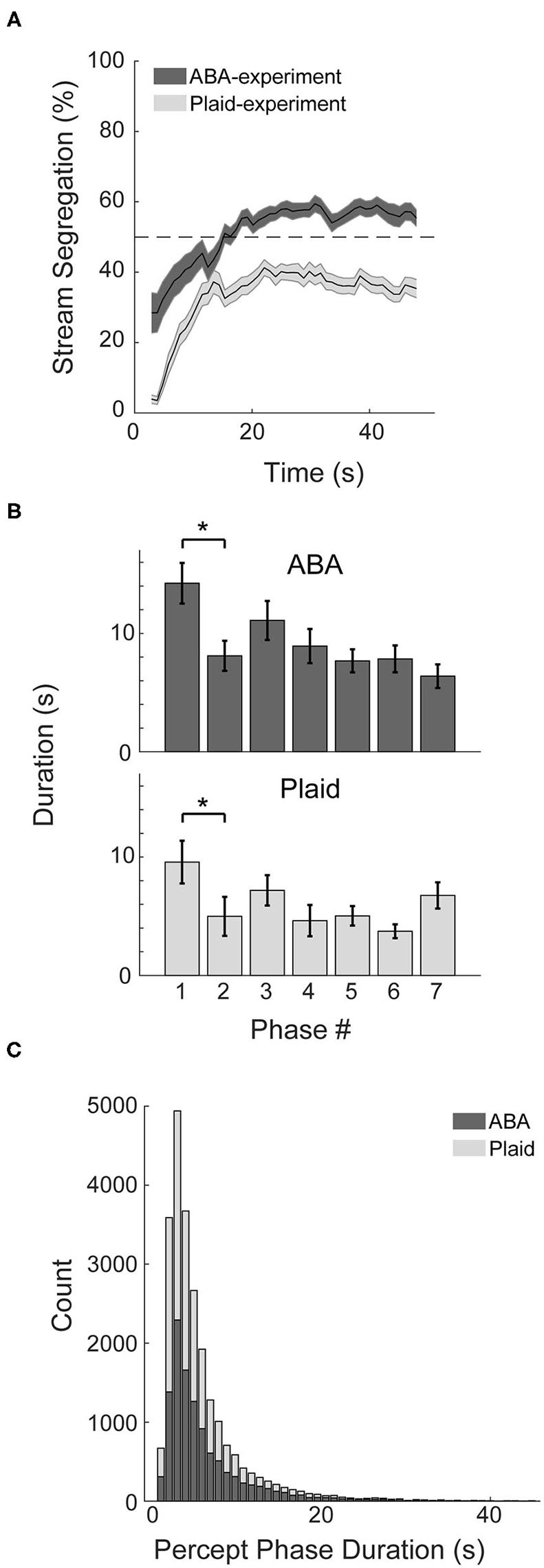
Characteristics of response patterns. **(A)** Percentage of stream segregation reported across the 48 s of a stimulus trial averaged across participants for the auditory ABA (dark gray) and visual Plaid (light gray) paradigms. Error bars correspond to SEM. **(B)** Duration of the first seven perceptual phases in sequence, averaged across subjects for each experiment. **(C)** Distribution of phase durations across both experiments. *p < 0.05.

Behavioral markers of bistable perception were observed in both the ABA- and Plaid-experiments. Participants typically reported an integrated percept initially, followed by convergence toward bistable perception that fluctuated between segregated and integrated over the course of the continuous 48 s presentation trials (Figure 2A). Following the initial 10 s, perceptual responses to the ABA stimuli averaged around 55% segregation, and ~38% segregation in response to the moving plaid stimuli. Additional characteristics of bistable perception were observed in the response patterns. The duration of the initial perceptual phase in both experiments was demonstrably longer than subsequent perceptual phase (Figure 2B), quantified with paired *t*-tests (ABA: *t*_27_ = 2.73, *p* < 0.05, *d* = 0.57; Plaid: *t*_24_ = 3.24, *p* < 0.01, *d* = 0.48), and the distribution of perceptual phases for both experiments approximated a logarithmic distribution with many short phases and less longer phases (Figure 2C).

### Effect of Distractors

**Figure 3:**
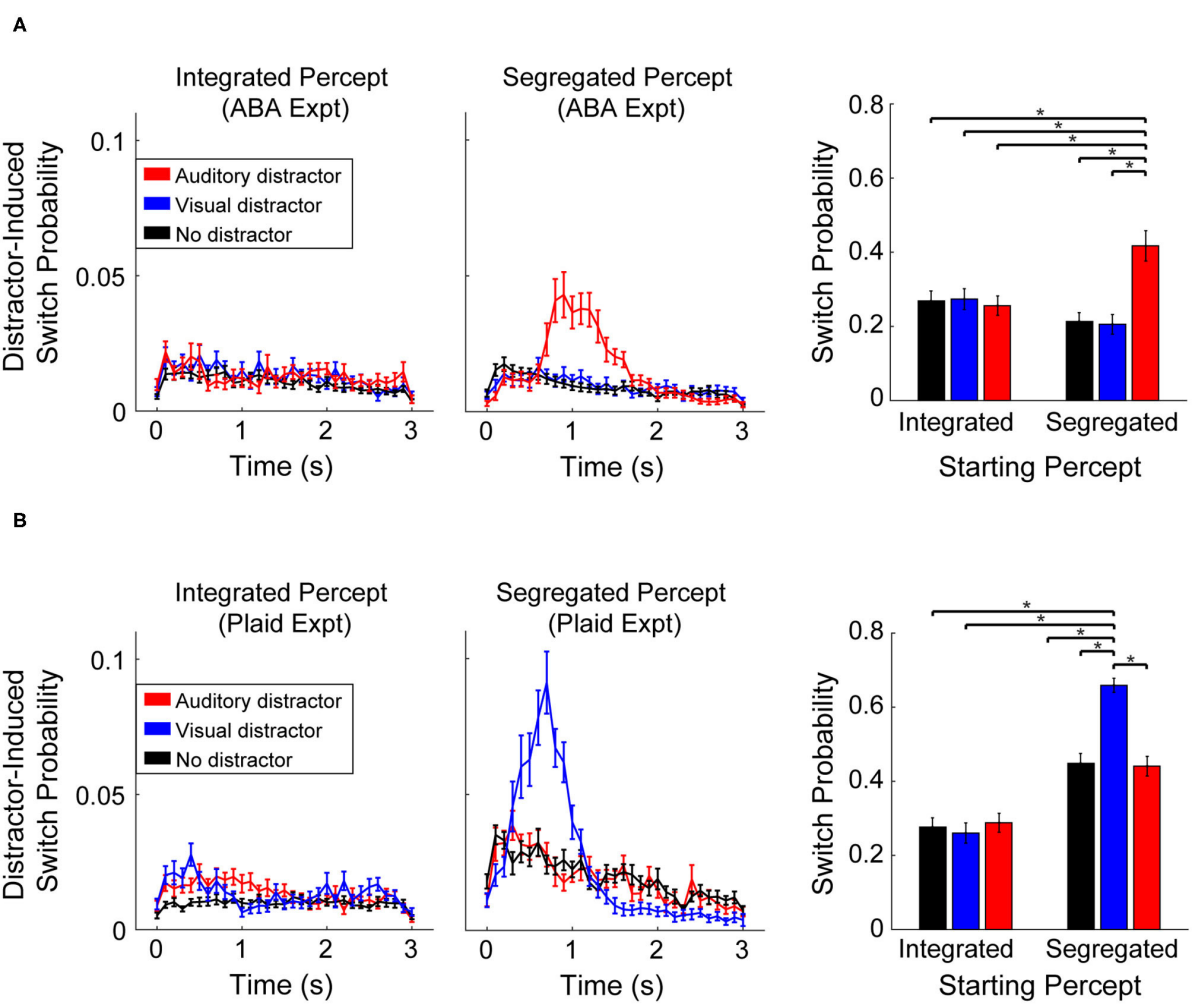
Probability of a switch following presentation of an auditory distractor (red), visual distractor (blue), or no-distractor (black). Line plots represent the probability time-course of a switch in perception (in 100 ms increments) following distractor presentation during an integrated (left column) or segregated percept, at distractor onset. Bar plots (right column) represent the cumulative probability of a perceptual switch following distractor (or no-distractor) presentation, measured over the time window from 300 to 2,000 ms (an extended time interval is shown here for illustrative purposes). Error bars correspond to SEM across participants. **(A)** Effect of distractor during the ABA-experiment. **(B)** Effect of distractors during the Plaid-experiment.

Participants indicated that perception of an integrated percept was unaffected by the presentation of auditory or visual distractors in both experiments. Illustrated in Figures 3A,B (left column), the probability of a switch following presentation of either distractor (Auditory-red line, Visual-blue line) was the same as when no-distractor was presented (black line). During segregated perception, however, participant responses revealed a strong effect of distractor within sensory modality. Auditory distractors led to a greater chance of a switch during segregation of the ABA stimuli (Figure 3A), while visual distractor presentation led to a greater chance of a switch during segregated perception of the moving plaid stimuli (Figure 3B). The mean switch time following a distractor was significantly slower for the ABA (1.3 s ± 0.37) than for the visual plaid (mean: 0.88 s ± 0.19), quantified with an unpaired *t*-test (t_58_ = 5.2, *p* < 0.001, *d* = 1.1). In both experiments, the alternate distractor (visual distractor during ABA-experiment, auditory distractor during Plaid-experiment) had no effect on perception compared to no-distractor.

The cumulative probability of a switch following distractor presentation (Figure 3, right column) was quantified with a repeated measures ANOVA with main effects of distractor-type and starting-percept. Results for the ABA experiment revealed a significant effect for distractor type (*F*_2, 68_ = 22.23, *p* < 0.001, $\eta_{\text{p}}^{2}$ = 0.40) and a significant interaction between distractor type and starting percept (*F*_2, 68_ = 37.5, *p* < 0.001, η^2^p = 0.524). There was no main effect for starting percept (*F*_1, 34_ = 0.77, *p* > 0.05). Comparison of individual conditions revealed that the probability of a switch following presentation of an auditory distractor was significantly greater (17%, on average) than all other conditions (*post-hoc* test: *t*_34_ > 4.6, *p* < 0.05; Bonferroni corrected). Statistical results for the Plaid-experiment showed a significant effect of distractor-type (*F*_2, 50_ = 37.55, *p* < 0.001, η^2^p = 0.60), starting percept (*F*_1, 25_ = 113.8, *p* < 0.001, η^2^p = 0.82), and a significant interaction between distractor-type and starting-percept (*F*_2, 50_ = 55.01, η^2^p = 0.69, *p* < 0.001). Comparison of individual conditions revealed that the probability of a switch following a visual distractor was significantly greater (32% on average) than all other conditions (*post-hoc* test: *t*_25_ > 8.3, *p* < 0.05; Bonferroni corrected). Note that the overall proportions of the two percepts (integrated vs. segregated) is unequal between the ABA- and Plaid-experiments (Figure 2A). As a result, the overall probability of a switch is relatively higher *within the analysis window* in the Plaid experiment for the segregated percept condition (Figure 3B, barplot).

## Discussion

In this set of experiments, we demonstrate that visual distractors have little effect on auditory stream segregation, and auditory distractors have little effect on visual stream segregation. In both experiments, however, the within-modality distractors significantly altered perception of segregation, effectively resetting perception. These results provide a valuable insight into the challenge necessary to maintain focus on a controlled process such as stream segregation, while also retaining a suitable level of awareness of other, competing sensory stimuli.

The utilization of the auditory ABA streaming and visual moving plaid as representative paradigms of perceptual segregation in each modality appears to be justified. Similar to observations made by Pressnitzer and Hupé (2006), while both paradigms elicited bistable perception, participants reported a segregated percept a smaller proportion of the time in response to the plaid compared to the ABA stimuli (Figure 2A). This characteristic also manifests as shorter phase durations (Figure 2B) and a higher overall probability of a switch within the analysis window (Figure 3A compared to Figure 3B) for the plaid stimulus. Despite these superficial differences, both paradigms demonstrated the tendency to build up from integrated to segregated over time, absent within-sensory modality distractors.

The complete ineffectiveness of the across-modality distractor to disrupt stream segregation strongly suggests a modality-specific generator for stimulus-induced perceptual switching. Denham et al. (2018) compared perceptual switching within subjects using the ABA streaming paradigm and an apparent motion paradigm. They concluded that despite within-subject consistency in spontaneous switch-rate between the two modalities, differences in the phase distributions indicates a distributed system across multiple brain regions, with similar but distinct processes for each modality. The results presented here are consistent with that conclusion.

Previous studies have shown disruption, or resetting of stream segregation due to prolonged gaps in the stimulus (Bregman, 1978; Cusack et al., 2004), noise bursts (Bregman, 1978), and shifts in spatial location and loudness (Rogers and Bregman 1998). Cusack et al. (2004) also demonstrated that a brief shift in attention is sufficient to reset segregated perception to integrated. It is important to note the distinction between these studies that observed streaming disruption based on sporadic, unpredictable stimuli, to those that demonstrate that periodic, predictable insertion of noise can in fact benefit speech segregation in normal hearing listeners (Miller and Licklider, 1950; Başkent and Chatterjee, 2010; Bologna et al., 2018). Further evidence provided by Rankin et al. (2017) shows that segregation can be enhanced via slight increases in the frequency of the B component of an otherwise stable ABA sequence (+2 semitones), indicating that the online streaming process goes in both directions, updating to confirm segregation, or disrupting to “reset” back to integrated. The difference between these studies and the results presented here highlights the ability of the auditory system to use preceding, or contextual information to build object segregation out of familiar, or enhanced pieces (speech segments or subtle changes in frequency content), and an inability to maintain segregation following unexpected disruption. In light of this literature, it is reasonable to speculate that if the current experiment had included a condition with regular (periodic) insertion of the distractor, the within-modality disruption effects would have been negated (Andreou et al., 2011).

Kondo et al. (2012b) showed that auditory stream segregation was reset from segregated to integrated upon initiation of self-induced head movement. This is an interesting result in the context of the current experiment. Disruption of segregation following self-initiated head movement may be interpreted as opposite to our results in that the motor input does disrupt auditory segregation whereas visual input does not. An alternate interpretation is that when a head movement occurs the configuration of elements in the auditory environment changes, and the buildup of segregation must reset. The second interpretation is consistent with the results of the current study and supports the hypothesis that the integrated percept is the default when circumstances demand a re-evaluation of the auditory scene.

Binocular rivalry studies in the visual system indicate that bistable perception is due to neural competition at multiple levels of the visual pathway, including the lateral geniculate nucleus, primary visual cortex, and the ventral pathway of the visual system (Leopold and Logothetis, 1996; Tong, 2001; Blake and Logothetis, 2002; Wunderlich et al., 2005). Support for the ventral pathway as a locus for stream segregation has also been observed in the auditory system (Curtu et al., 2019; Higgins et al., 2020), and this conclusion is further supported by computational modeling that most accurately describes bistable perception as the result of competing levels of adaptation, inhibition, and noise across three levels of hierarchical processing (Little et al., 2020). The resulting hypothesis is that segregation emerges to varying degrees of the ascending sensory system, and is most prominent at later levels of the ventral pathway.

As information ascends through the central nervous system there is an integrative multi-sensory process based on spatial and temporal coincidence. If there is enough similarity across these dimensions to be informative, the evidence indicates that multi-sensory facilitation occurs, resulting in increased reaction time (Schröger and Widmann, 1998), speech comprehension (Callan et al., 2004), and visual perception (McDonald et al., 2000; Frassinetti et al., 2002; Lippert et al., 2007), for example. In this multisensory context, the results of the experiments in this study can be summarized: within-modality distractors provide a bottom-up disruption of the segregated percept due to representational overlap at a stage of processing where segregated streams have been built up based on predictable patterns. The distractor breaks those patterns and resets perception to the default. Due to the experimental design, presentation of the alternate-distractor did not align temporally with the stimulus, and therefore did not overlap in representational space with the segregated streams, and had no impact on stream segregation in either domain.

## Summary

Two experiments testing bistable perception were carried out, one in the auditory domain and one in the visual domain. While participants indicated their perception, auditory and visual distractors were intermittently introduced to the stimuli. In each experiment the within-modality distractor effectively *reset* the segregated percept back to integrated, while the alternate-modality distractor had no impact on perception. Results support the hypothesis that the cognitive mechanism that modulates perception is domain specific rather than global.

## Data Availability Statement

The original contributions presented in the study are publicly available. This data can be found here: https://osf.io/tbdxr/?view_only=b682b00298c84f9eba3dfdfee39125b8.

## Ethics Statement

The studies involving human participants were reviewed and approved by University of Nevada, Las Vegas, Internal Review Board. The patients/participants provided their written informed consent to participate in this study.

## Author Contributions

NH and AM designed study, conducted experiment, analyzed data, and contributed to manuscript. BY conducted experiment and analyzed data. DL and JN-B designed study. ME contributed to manuscript. JS designed study, contributed to manuscript, and analysis. All authors contributed to the article and approved the submitted version.

## Funding

This work was supported by Office of Naval Research: N00014-16-1-2879.

## Conflict of Interest

The authors declare that the research was conducted in the absence of any commercial or financial relationships that could be construed as a potential conflict of interest.

## Publisher's Note

All claims expressed in this article are solely those of the authors and do not necessarily represent those of their affiliated organizations, or those of the publisher, the editors and the reviewers. Any product that may be evaluated in this article, or claim that may be made by its manufacturer, is not guaranteed or endorsed by the publisher.
